# Advances in Gastric Cancer Research: Insights Into Carcinogenesis, the Tumor Microenvironment, Metastasis, and Factors Influencing Prognosis

**DOI:** 10.1002/ags3.70195

**Published:** 2026-04-27

**Authors:** Hideo Baba, Takatsugu Ishimoto, Yoshifumi Baba, Hiromitsu Hayashi, Masaaki Iwatsuki

**Affiliations:** ^1^ Department of Gastroenterological Surgery Kumamoto University Kumamoto Japan; ^2^ The Chemo‐Sero‐Therapeutic Reaserch Institute Kumamoto Japan; ^3^ Division of Carcinogenesis The Cancer Institute, Japanese Foundation for Cancer Research Tokyo Japan; ^4^ Department of Gastrointestinal Surgery, Graduate School of Medicine The University of Tokyo Tokyo Japan

**Keywords:** CAF, cancer progression, carcinogenesis, gastric cancer, immunotherapy, microbiota, tumor microenvironment

## Abstract

**Background:**

The Department of Gastroenterological Surgery at Kumamoto University has maintained a commitment to integrating cutting‐edge clinical practice with fundamental research, particularly concerning malignant diseases of the digestive tract. This comprehensive review aimed to synthesize and consolidate the key clinical and translational research achievements published by our department, primarily focusing on gastric cancer (GC) and the pathophysiological mechanisms that drive its progression.

**Methods:**

We systematically reviewed the most impactful English‐language original research papers from our institution, since 2005. The identified studies span critical areas including molecular carcinogenesis, tumor microenvironment (TME) components, and mechanisms of systemic metastasis and drug resistance, and so on.

**Results:**

Our accumulated research has yielded significant insights into the molecular basis of GC. A core area of contribution includes the genetic and epigenetic changes during carcinogenesis of gastric cancer or Gastric Adenocarcinoma and Proximal Polyposis of the Stomach (GAPPS), the tumor microenvironment (TME) especially focusing on Cancer‐Associated Fibroblasts (CAFs), a factor promoting peritoneal dissemination of ECM‐related genes and gut microbiota. Furthermore, our translational work has explored mechanisms of resistance to chemotherapeutic agents.

**Conclusion:**

The collective research from our department represents a crucial contribution to the global understanding of gastric cancer pathogenesis, progression, and clinical management. These findings underscore our commitment to translating basic scientific discoveries into actionable strategies that advance individualized therapy, ultimately improving the long‐term prognosis for patients with digestive malignancies.

## Abstract 2

This review article is the summary of JSGS Science of the Year 2023, which has been given to Hideo Baba, the first author of this manuscript, and has focused mainly on the molecular basis of GC. A core area of contribution includes the genetic and epigenetic changes during carcinogenesis of gastric cancer or Gastric Adenocarcinoma and Proximal Polyposis of the Stomach (GAPPS), tumor microenvironment (TME) especially focusing on Cancer‐Associated Fibroblasts (CAFs), a factor promoting peritoneal dissemination of ECM‐related genes, and gut microbiota.

## 1 Introduction

**FIGURE 1:**
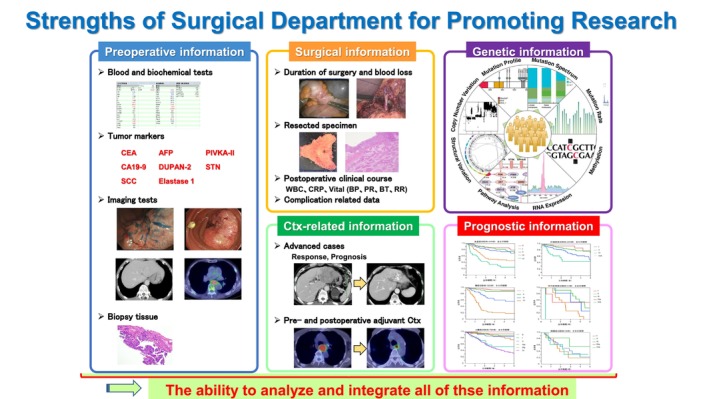
The advantages of surgeons to do both clinical and basic research work. Surgeons have access to a wealth of information. They possess extensive data from before surgery, such as blood and biochemical test results, tumor markers, and imaging. They also have a great deal of information related to the surgery itself, including operative time, blood loss, and pathological findings from the resected specimen. Additional information includes postoperative clinical progress, complications, imaging tests before and after chemotherapy, genetic data from resected specimens and biopsy samples, and prognostic information. By analyzing and integrating this information, surgeons can generate a lot of evidence, which is why they are considered to have a great advantage when it comes to conducting research.

In Japan, the number of surgeons has recently been declining, with a particularly noticeable decrease in gastrointestinal surgeons. Surgeons spend a considerable amount of time on clinical duties, limiting the time they can dedicate to research. As a result, the combination of a shortage of manpower and lack of time has led to a continuous decline in research papers from surgical departments. However, surgeons have access to a wealth of information. They possess extensive data from before surgery, such as blood and biochemical test results, tumor markers, and imaging. They also have a great deal of information related to the surgery itself, including operative time, blood loss, and pathological findings from the resected specimen. Additional information includes postoperative clinical progress, complications, imaging tests before and after chemotherapy, genetic data from resected specimens and biopsy samples, and prognostic information. By analyzing and integrating this information, surgeons can generate a lot of evidence, which is why they are considered to have a great advantage when it comes to conducting research (Figure 1).

At Kumamoto University's Department of Gastroenterological Surgery, we have advanced research on various gastrointestinal cancers from multiple perspectives, including stem cells, epigenetics, metabolism, the tumor microenvironment, gut microbiota, chemoprevention, genomics, and tumor immunology. In this review, we have focused on gastric cancer and summarized our recent research findings.

Gastric cancer (GC) remains a significant global health burden, characterized by high incidence and mortality rates. Its pathogenesis is a complex, multifactorial process involving distinct stages of carcinogenesis, local progression, and distant metastasis, all influenced by a dynamic interplay between tumor cells and their microenvironment. The tumor microenvironment (TME), particularly the role of cancer‐associated fibroblasts (CAFs), has emerged as a critical determinant of GC behavior. Furthermore, epigenetic alterations, including DNA methylation changes like Long Interspersed Nucleotide Element‐1 (LINE‐1) hypomethylation and microRNA dysregulation, are increasingly recognized for their contributions to tumor initiation and advancement. This review provides a comprehensive overview of the current understanding of GC, focusing on the molecular underpinnings of its development, spread, and the factors dictating patient prognosis. Therapeutic challenges, notably chemoresistance, are also explored, with an emphasis on mechanisms involving CAF‐derived extracellular vesicles or microbiome infection such as *Fusobacterium nucleatum* ( *F. nucleatum* ). Throughout this review, significant research contributions from our laboratory at Kumamoto University are integrated, highlighting our impact on elucidating these complex processes and identifying novel therapeutic and prognostic avenues. In this review, we have also added a part of our recent results of gastroenterological research topics other than gastric cancer.

## 2 Key Genetic and Epigenetic Alterations

Gastric carcinogenesis is driven by a complex spectrum of genetic mutations [1, 2, 3]. Frequently mutated genes include *TP53*, a critical tumor suppressor involved in cell cycle control and apoptosis; *CDH1*, which encodes E‐cadherin, essential for cell adhesion and mutated particularly in diffuse‐type GC; and *ARID1A*, a component of the SWI/SNF chromatin remodeling complex. In addition to somatic mutations, epigenetic modifications play a pivotal role in shaping the cancer genome and transcriptome [4]. These heritable changes in gene expression occur without alterations to the DNA sequence itself. Key epigenetic mechanisms implicated in GC include DNA methylation, histone modifications, and the activity of non‐coding RNAs. DNA methylation typically involves the addition of a methyl group to cytosine residues within CpG dinucleotides. In cancer, this process is frequently dysregulated, leading to hypermethylation of CpG islands in the promoter regions of tumor suppressor genes, resulting in their silencing. Conversely, global DNA hypomethylation, particularly in repetitive DNA sequences, is also a common feature of cancer cells, contributing to genomic instability.

One such repetitive element, LINE‐1, constitutes a significant portion of the human genome (approximately 17%), and its methylation level is considered a surrogate marker for global DNA methylation [5, 6]. Our group has provided important insights into the role of LINE‐1 hypomethylation in GC [7]. Using bisulfite pyrosequencing, we quantified LINE‐1 methylation levels in resected GC specimens and observed significantly lower methylation in tumor tissues compared to matched normal gastric mucosa. Crucially, this tumoral LINE‐1 hypomethylation was significantly associated with shorter overall survival, suggesting its potential as a prognostic biomarker. Furthermore, LINE‐1 hypomethylation has been linked to genomic instability and, in other cancers like esophageal squamous cell carcinoma (ESCC) [8], to environmental exposures such as tobacco smoking [9]. This suggests that LINE‐1 hypomethylation may not merely be a passive marker but an active contributor to carcinogenesis. Environmental carcinogens can induce epigenetic alterations, and genomic instability is a recognized hallmark of cancer. Therefore, LINE‐1 hypomethylation could represent a mechanistic bridge between certain carcinogenic exposures and the development of GC by fostering a genomically unstable environment that is more susceptible to acquiring further mutations and driving cancer progression [10]. This implies that monitoring LINE‐1 methylation could be valuable for risk assessment, and interventions aimed at stabilizing the epigenome might hold preventative potential.

## 3 Gastric Cancer Carcinogenesis

Cancer stem cells (CSCs) are known to be involved in carcinogenesis, cancer progression, chemotherapy resistance, and the development of metastases [11, 12]. There are accumulating evidence regarding gastric CSCs, current insights into the immune microenvironment, and therapeutic targets [13, 14]. We revealed that a CD44 variant (CD44v), prevalent in cancer stem‐like cells, interacts with the xCT transporter, crucial for maintaining intracellular reduced glutathione (GSH) levels [15]. High CD44 expression in human gastrointestinal cancer cells correlated with increased GSH synthesis and enhanced defense against reactive oxygen species (ROS). Conversely, CD44 ablation led to xCT loss from the cell surface, suppressed tumor growth in mice through accumulating ROS‐dependent p38(MAPK) activation and p21(CIP1/WAF1) upregulation. These findings underscore the critical role of CD44v in regulating cellular redox balance and promoting tumor progression.

Cancer development is often preceded by the appearance of preneoplastic lesions. In gastric carcinogenesis, chronic inflammation and histopathologic progression of the stomach epithelium lead to the development of metaplasia and eventually adenocarcinoma. The CD44v‐xCT system has a functional role in the development of spasmolytic polypeptide‐expressing metaplasia as a preneoplastic lesion [16]. Chronic inflammation with *Helicobacter pylori (H. pylori*) infection, which is the main risk factor for the development of gastric cancer, is implicated in CD44 overexpression through miR‐328 suppression in the gastric mucosa [17]. Infection with *H. pylori* , CD44 overexpression, especially that of variant 9 (CD44v9), has also been implicated in the local inflammatory response and metaplasia‐carcinoma sequence in humans. Macrophage‐derived ROS suppress miR‐328 targeting CD44 in cancer cells and promote redox adaptation through CD44 induction. Supporting this finding, induction of CD44 expression by miR‐328 inhibitor led to promotion of cancer cell growth. Moreover, we have examined the significance of CD44(+) tumor cells in tumor development using circulating tumor cells (CTCs) from 42 advanced gastric cancer patients [18]. Patient peripheral blood mononuclear cells (PBMCs) were injected into immunodeficient mice, establishing nine human‐origin tumor‐like structures from six patients. Notably, one tumor developed from CD45(−) PBMCs, reflecting “authorized” CTCs, while others originated from CD45(+) fractions. These tumor‐initiating cells expressed EpCAM, CEA, and notably, varying levels of CD44, predominantly CD44v8–10. CD44(+/high) cells exhibited higher tumorigenicity and xCT expression. These findings confirm the presence of tumor‐initiating cells in peripheral blood of advanced gastric cancer patients, suggesting potential therapeutic targets and research tools.

Our recent experience with a few families of GAPPS, a rare, inherited gastric cancer syndrome, drove us to further focus on elucidating the mechanisms of carcinogenesis of GAPPS [19]. GAPPS is in general caused by a point mutation in the *APC* gene's promoter 1B region [20].

**FIGURE 2:**
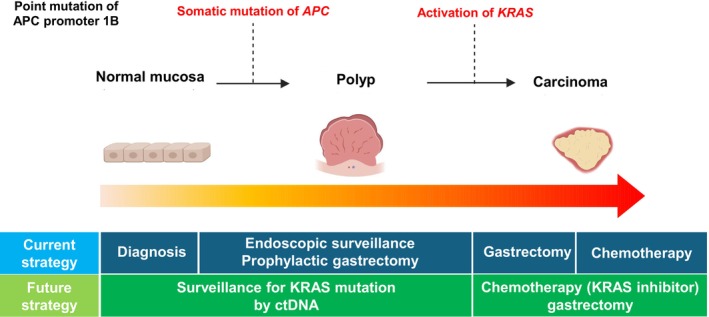
The landscape of carcinogenesis in GAPPS. Compared with normal mucosa, somatic mutations in APC occur in polyps, and additional KRAS mutations arise in carcinomas. The current treatment strategies are mainly endoscopic surveillance and prophylactic resection; however, if surveillance using ctDNA and other liquid biopsy methods becomes feasible in the future, therapeutic interventions could be implemented at a more appropriate timing.

We energetically investigated the genomic and transcriptomic landscape of GAPPS, an autosomal dominant syndrome with a high tendency for gastric adenocarcinoma [21, 22]. We analyzed 54 samples from seven GAPPS patients, encompassing normal mucosa, polyps, and carcinomas, using whole‐exome and RNA sequencing. Key findings reveal that somatic *APC* mutations occur in both carcinoma and polyp samples, while *KRAS* mutations are specific to carcinomas. The co‐occurrence of *APC* and *KRAS* mutations was recurrently observed in carcinomas across cases and within subclones. This co‐occurrence is suggested to be almost essential for GAPPS‐associated carcinoma (Figure 2). *KRAS* mutations could serve as a potential biomarker for early detection and determining resection timing in GAPPS carcinogenesis. The study highlights distinctive mutational processes in GAPPS compared to sporadic gastric cancer, where *APC* and *KRAS* co‐occurrence is rare and *TP53* mutations are absent.

## 4 Tumor Microenvironment and Cancer‐Associated Fibroblasts (CAFs)

Cancer cells are not passive inhabitants within a tumor; they actively manipulate their surrounding microenvironment (TME) to support their growth, survival, and spread [23, 24]. This manipulation is a crucial aspect of cancer progression. Here's a brief overview of how they do this:

- Remodeling the Extracellular Matrix (ECM): Cancer cells secrete enzymes like matrix metalloproteinases (MMPs) that break down and remodel the ECM. This creates pathways for invasion and metastasis and also releases growth factors that further promote tumor growth.
- Educating Immune Cells: Instead of being eliminated by the immune system, cancer cells can “re‐educate” immune cells within the TME. They release molecules that suppress anti‐tumor immune responses and promote the development of pro‐tumor immune cells, such as M2 macrophages and regulatory T cells (Tregs)
- Inducing Angiogenesis: To fuel their rapid growth, cancer cells stimulate the formation of new blood vessels (angiogenesis). They release pro‐angiogenic factors like Vascular Endothelial Growth Factor (VEGF), ensuring a constant supply of oxygen and nutrients.
- Altering Metabolism: Cancer cells often exhibit altered metabolism (e.g., increased glycolysis, known as the Warburg effect). This not only provides them with energy and building blocks but also creates an acidic TME that can suppress immune cells and promote invasiveness.
- Recruiting Stromal Cells: Cancer cells actively recruit and activate various stromal cells, including fibroblasts (which become cancer‐associated fibroblasts or CAFs) and mesenchymal stem cells. These cells then contribute to ECM remodeling, secrete growth factors, and support tumor growth and metastasis.
- Promoting Inflammation: While chronic inflammation can enhance cancer initiation, cancer cells also promote an inflammatory environment within the TME. This inflammation can provide growth factors, promote angiogenesis, and suppress anti‐tumor immunity.

In essence, cancer cells act as orchestrators, transforming the normal tissue microenvironment into a supportive niche that facilitates their own malignant behavior. Understanding these interactions is vital for developing effective cancer therapies.

Once initiated, gastric cancer progresses through local invasion into surrounding tissues, and this process is heavily influenced by complex interactions between cancer cells and various components of the TME [25]. CAFs, in particular, play a central role in orchestrating this invasive behavior and metastasis of gastric cancer and are considered as a potential therapeutic target in the TME [26]. CAFs are a dominant cell type within the stroma of many solid tumors, including GC, and are characterized by their heterogeneity and plasticity. They can originate from various precursor cells, including resident tissue fibroblasts, bone marrow‐derived mesenchymal stem cells, or through processes like epithelial‐mesenchymal transition (EMT) or endothelial‐mesenchymal transition (EndMT). To understand their identities, CAFs from gastric cancer tissues and adjacent non‐cancer fibroblasts (NFs) have been isolated and we also developed the novel tools and protocols related with CAFs to facilitate further research into CAFs [27]. Moreover, we developed a novel tdTomato transgenic mouse model to visualize FAP‐positive CAFs activated fibroblasts [28]. Coculturing a mouse gastric cancer cell line with fibroblasts derived from FAP‐tdTomato transgenic mice showed elevated tdTomato expression in the cocultured fibroblasts, indicating the utility for further investigation.

We investigated CAFs in diffuse‐type gastric cancers (DGCs) and found that many genes associated with transforming growth factor beta 1 (TGFβ1) activity were upregulated in CAFs. We observed increased expression of Rhomboid 5 homolog 2 (RHBDF2), which regulates TGFβ1 signaling. Expression of RHBDF2 in fibroblasts is induced by inflammatory cytokines secreted by DGCs [29]. RHBDF2 promotes cleavage of TGFBR1 by activating TACE and motility of CAFs in response to TGFβ1. These highly motile CAFs induce DGCs to invade extracellular matrix and lymphatic vessels in nude mice.

In pancreatic cancer, which is abundant in fibrous tissue like gastric cancer, CAFs repurpose lactate produced by highly glycolytic cancer cells as an energy source. This metabolic shift, driven by glucose scarcity, enables CAFs to proliferate via the TCA cycle and establish an immunosuppressive microenvironment [30]. They upregulate IL‐6 expression, which, along with lactate, suppresses cytotoxic immune cell activity. Targeting lactate dehydrogenase A (LDHA), which regulates lactate production, with inhibitors like FX11, reduced tumor growth and improved anti‐tumor immunity in CAF‐rich pancreatic cancer models. These findings highlight that lactate‐mediated crosstalk between tumor cells, CAFs, and immune cells as a potential therapeutic target.

We subsequently studied the specific molecular mechanisms through which CAFs exert their pro‐tumorigenic effects, including promoting drug resistance and immune escape. The expression of programmed death‐ligand 1 (PD‐L1) in tumor cells contributes to tumor immune escape. We had investigated the precise mechanism regulating PD‐L1 expression in gastric cancer cells. Specifically, CAFs‐derived IL‐8 upregulates PD‐L1 expression in gastric cancer through the NF‐κB pathway [31]. In addition to IL‐8 from CAFs, extracellular vesicles (EVs) from CAFs promote gastric cancer progression and drug resistance [32]. This study elucidates a novel molecular mechanism through which Annexin A6 in CAF‐EVs activates FAK‐YAP by stabilizing β1 integrin at the cell surface of gastric cancer cells and subsequently induces drug resistance.

Given the significant role of CAFs in TME, we explored strategies to target them to improve treatment outcomes. We investigated how stromal reprogramming can enhance cancer immunotherapy, particularly in fibrotic tumors. We found that PDGFC and D correlate with poor gastric cancer prognosis, and PDGFRβ is highly expressed in diffuse‐type gastric cancer stroma [33]. PDGF stimulation in CAFs increased CXCL1, 3, 5, and 8, recruiting PMN‐MDSCs, leading to anti‐PD‐1 resistance in fibrotic tumors. The combination of PDGFRα/β blockade and anti‐PD‐1 treatment synergistically suppressed fibrotic tumor growth. These findings highlight stromal targeting as a promising strategy to boost immunotherapy in fibrotic cancers.

**FIGURE 3:**
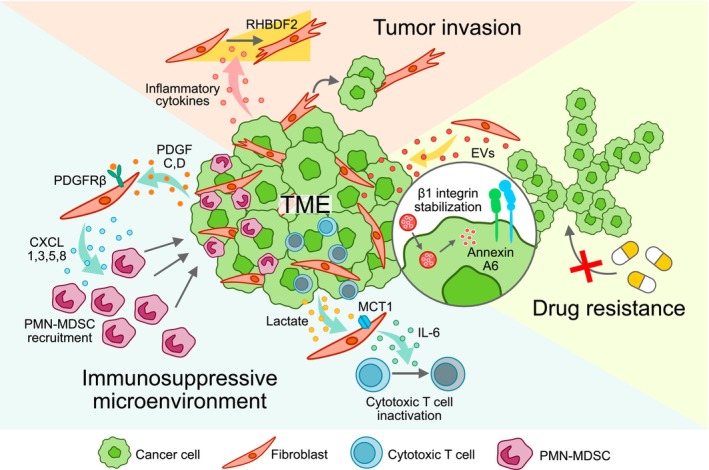
The interaction of cancer cells and tumor microenvironment. The progression of the tumor includes not only cancer cells but several cell types in the tumor microenvironment. Among the tumor microenvironment, the role of CAFs is important. CAFs exert the roles of tumor invasion, drug resistance, and immunosuppressive microenvironment.

The above‐mentioned role of TME in relation to tumor invasion, drug resistance, and immunosuppressive microenvironment is summarized in Figure 3.

## 5 The Role of Chronic Inflammation and Microbial Factors

Chronic inflammation is a well‐established risk factor for various cancers. The most significant inflammatory trigger in the stomach is chronic infection with *H. pylori* . This bacterium colonizes the gastric mucosa and can induce a persistent inflammatory response, leading to gastritis, atrophy, intestinal metaplasia, dysplasia, and eventually carcinoma [34]. Key virulence factors of *H. pylori* , such as CagA (cytotoxin‐associated gene A) and VacA (vacuolating cytotoxin A), contribute to its pathogenicity by disrupting cellular signaling pathways, promoting cell proliferation, and inducing DNA damage. Chronic gastritis, whether due to *H. pylori* or autoimmune conditions, can lead to malignancy through several mechanisms: the causative agent itself may be genotoxic; substances released from inflammatory cells can be genotoxic; inflammation‐induced cell death can trigger compensatory proliferation, increasing the risk of mutations.

Epstein–Barr Virus (EBV)‐associated gastric cancer (EBVaGC) represents a distinct molecular subtype, accounting for approximately 10% of GC cases [2]. EBVaGC is characterized by unique epigenetic features, including global CpG island hypermethylation and specific viral gene expression patterns.

Dysbiosis, or an imbalance in the microbial community, can contribute to chronic inflammation, modulate host immune responses, and even produce metabolites that affect cancer cell behavior [35].

The human intestinal microbiome is vast, encompassing at least 100 trillion microorganisms that can influence host immunity and disease conditions, including cancer. Our research has focused on *F. nucleatum* , which primarily inhabits the oral cavity, causes periodontal disease, and has been implicated in the development of human cancers. We quantified *F. nucleatum* DNA in 325 resected esophageal cancer specimens, finding its presence in 23% of cases and significantly more abundant in tumor tissue than normal mucosa [36]. *F. nucleatum* positivity was associated with advanced tumor stage and, importantly, with reducedcancer‐specific survival (HR = 1.78, *p* = 0.031). KEGG analysis highlighted “cytokine‐cytokine receptor interaction” as a key pathway, with CCL20 chemokine validated. These findings suggest *F. nucleatum* may serve as a potential prognostic biomarker and may contribute to aggressive esophageal tumor behavior through chemokine‐mediated mechanisms.

*F. nucleatum* has been shown to infiltrate ESCC cells and modulate autophagy, specifically by altering LC3 and ATG7 expression and autophagosome formation, leading to resistance against common chemotherapeutic agents (5‐FU, CDDP, Docetaxel) [37]. Importantly, knockdown of ATG7 reversed this chemoresistant phenotype, suggesting that targeting *F. nucleatum* during chemotherapy may enhance therapeutic efficacy in ESCC. We confirmed that co‐culture of *F. nucleatum* and ESCC cells exhibited enhanced growth, NF‐κB activation, and overexpression of NOD1 and phosphorylated RIPK2. In xenograft models, *F. nucleatum* ‐treated cells showed accelerated tumor growth with NF‐κB activation [38].

We further investigated the impact of *F. nucleatum* on esophagogastric junction (EGJ) and gastric cancers using two patient cohorts (Japan and USA) and cell line/xenograft models. We found that *F. nucleatum* ‐positive patients had significantly shorter overall survival. In vitro and in vivo experiments showed *F. nucleatum* promotes gastric cancer cell proliferation and NF‐κB pathway activation, leading to tumor progression [39].

Our research has also broadened to investigate the microbiome's role in various gastrointestinal cancers, which includes hepatobiliary‐pancreatic cancers [40], colorectal cancer, and colorectal cancer liver metastasis [41]. Another study investigated whether preoperative serum iron status, which can influence microbiota, was associated with prognosis after colorectal cancer resection [42]. Overall, this research consistently explores the intricate relationship between the gut microbiome and various gastrointestinal cancers, aiming for a better understanding of how the gut microbiota influences tumor development and progression. The integration of microbiology into molecular pathological epidemiology (MPE) models is also highlighted as a way to analyze tumor‐immune‐microbiome interactions.

## 6 EMT And Cancer Metastasis, Focusing on Peritoneal Dissemination

### 6.1 EMT

We have extensively investigated the critical role of Epithelial‐Mesenchymal Transition (EMT) in various cancers. EMT is a crucial biological process that plays a significant role in cancer metastasis, which is a complex cellular program where epithelial cells undergo a series of molecular and morphological changes to acquire characteristics of mesenchymal cells. Mesenchymal cells are typically more migratory, invasive, and less adherent [43].

During the process of EMT, the following phenomenon is included: (1) loss of cell–cell adhesion caused by a loss of E‐cadherin, (2) loss of cell polarity like as a distinct apical‐basal polarity, (3) acquisition of migratory and invasive properties, (4) increased resistance to apoptosis, (5) acquisition of stem‐cell like properties.

EMT significantly contributes to the metastatic cascade through several steps such as detachment from the primary tumor, invasion, intravasation, circulation and survival, extravasation, colonization of which a reverse process called Mesenchymal‐Epithelial Transition (MET), re‐acquiring epithelial characteristics to proliferate and form a macroscopic colony.

EMT plays a critical role in the progression of gastric cancer. Vimentin (VIM) expression, an indicator of mesenchymal characteristics, was found in bone marrow of gastric cancer patients, suggesting that gastric cancer cells undergo EMT to survive in this environment [44]. MicroRNA‐200b (miR‐200b) is shown to regulate cell proliferation, invasion, and migration by directly targeting ZEB2, thereby suppressing EMT in gastric carcinoma.

Other than gastric cancer, high CD44 standard isoform (CD44s) expression was associated with an EMT expression profile and intrahepatic dissemination of HCC after local ablation therapy [45]. Lysyl oxidase (LOX) induces EMT and predicts intrahepatic metastasis of HCC, with bioinformatic analysis suggesting that HIF‐1alpha or hypoxia regulates LOX expression and induces EMT [46].

Hyperglycemia induces metabolic reprogramming to a glycolytic phenotype and promotes EMT via the YAP/TAZ‐Hedgehog signaling axis in pancreatic cancer [47]. Knockdown or inhibition of YAP/TAZ can abolish EMT, chemoresistance, and a favorable tumor microenvironment even under hyperglycemic conditions.

Overall, these studies demonstrate the profound involvement of EMT in the progression, metastasis, and prognosis of various cancers, and identify key molecular players and signaling pathways that influence this critical process, offering potential targets for therapeutic intervention.

### 6.2 Peritoneal Dissemination

Peritoneal dissemination (PD) occurs when GC cells scatter from the primary tumor, survive in the peritoneal fluid, and then implant and grow on the peritoneal surfaces [48, 49]. It is the most frequent metastasis in GC and is associated with a dismal prognosis due to the lack of effective systemic therapies. Multi‐step processes are required for the PD development:

- Detachment from the primary tumor and invasion through the gastric wall: This initial step requires cancer cells to lose cell–cell adhesion, often mediated by the downregulation or functional inactivation of E‐cadherin. Increased cell motility and invasive capacity are driven by factors such as activated Rho GTPases (e.g., RhoA, Rac, cdc42), which reorganize the actin cytoskeleton, and ADP‐ribosylation factor‐like 4C (*ARL4C*), a downstream effector of EGF and Wnt signaling. Degradation of the ECM by MMPs, including MMP‐7, MMP‐2, MMP‐9, and MMP‐14, is also crucial for cells to penetrate the gastric serosa and enter the peritoneal cavity. The EMT program is heavily implicated in this detachment and invasion phase.
- Survival and adaptation in the peritoneal cavity microenvironment: Once disseminated in the peritoneal cavity, cancer cells face a harsh environment characterized by lack of adhesion to ECM (anoikis‐inducing conditions), nutrient deprivation, and hypoxia. To survive and proliferate, cells must develop anoikis resistance. This is often achieved through alterations in integrin signaling, activation of survival pathways such as PI3K/Akt and FAK (Focal Adhesion Kinase), and metabolic reprogramming. Hypoxia‐Inducible Factor 1‐alpha (HIF1α) plays a key role in adaptation to hypoxic conditions and can also promote EMT and anoikis resistance, partly by inducing factors like Angiopoietin‐like 4 (*ANGPTL4*). The CXCL12/CXCR4 chemokine axis has also been implicated in promoting anoikis resistance and PD.
- Attachment to peritoneal mesothelial cells and tumor growth: Surviving cancer cells must then adhere to the mesothelial lining of the peritoneum. This attachment is mediated by specific cell adhesion molecules and integrins, such as integrin α3β1, which binds to ECM components like laminin‐5. Following adhesion, cancer cells invade the submesothelial tissue, proliferate, and induce angiogenesis (the formation of new blood vessels) to support the growth of peritoneal nodules. Vascular Endothelial Growth Factor (VEGF) is a key pro‐angiogenic factor involved in this stage.

Our recent study on peritoneal dissemination in gastric cancer can be summarized as follows: indicating a research flow from understanding the microenvironment and cellular mechanisms to exploring therapeutic interventions:

Recent studies have identified PLOD2 and DDR2 as key regulators involved in peritoneal dissemination. PLOD2, a collagen‐modifying enzyme, promotes tumor progression and peritoneal dissemination by remodeling the extracellular matrix [50], while DDR2, a receptor tyrosine kinase, was highlighted through integrated molecular profiling as a potential driver of metastatic spread [51].

Inflammation‐driven senescence‐associated secretory phenotype (SASP) in cancer‐associated fibroblasts enhances peritoneal dissemination [52]. In the tumor microenvironment, senescent non‐malignant cells, including cancer‐associated fibroblasts (CAFs), exhibit SASP under stress conditions. This SASP is shown to promote cancer progression and chemoresistance, thereby enhancing peritoneal dissemination.

Malignant ascites, a common feature of peritoneal dissemination, contain various factors and cell populations. We demonstrated that proinflammatory cytokines induce the activation of NF‐kB signaling and cellular senescence by promoting ROS accumulation in CAFs. Subsequently, the expression of SASP factors, particularly IL‐6, is maintained at a high level through the demethylation of H3K27me3 marks by EZH2 downregulation, resulting in enhanced peritoneal tumor formation through JAK/STAT3 signaling. Moreover, single cell analysis using ascites from gastric cancer patients revealed that five CD45(−)/EpCAM(−) cell subpopulations, with mesothelial cells exhibiting mesenchymal features being a primary source of chemokines that recruit immunosuppressive myeloid cells [53]. These mesothelial cells, undergoing mesothelial‐mesenchymal transition (MMT), also highly express ECM‐related genes like tenascin‐C (TNC), which enhances metastatic colonization. This research highlights that mesothelial cells, particularly those adopting mesenchymal features, contribute significantly to shaping a protumorigenic microenvironment, which subsequently enhances peritoneal dissemination.

## 7 Factors Influencing Clinical Outcome

Accurate prognostication is crucial for guiding treatment decisions, counseling patients, and designing clinical trials. Prognosis in GC is influenced by a combination of traditional clinicopathological factors and an expanding array of molecular biomarkers. The most widely used prognostic system for GC is the TNM staging system. Other established pathological factors include histological grade (degree of differentiation), tumor location within the stomach, and histological subtype according to classifications like Lauren (intestinal vs. diffuse). As previously mentioned, specific subtypes such as scirrhous gastric cancer are associated with a particularly aggressive clinical course and poor outcome. The search for molecular biomarkers aimed to refine prognostication beyond traditional factors and to identify patients who might benefit from specific therapies.

### 7.1 Nutritional Status (CONUT Score)

Systemic inflammation and nutritional status are increasingly recognized as important determinants of cancer patient outcomes. We investigated the prognostic utility of the preoperative Controlling Nutritional Status (CONUT) score in GC patients undergoing curative resection. The CONUT score is calculated based on serum albumin levels, total lymphocyte count, and total cholesterol levels [54]. In our retrospective study of 416 patients, we found that a high CONUT score (CONUT‐high: ≥ 4), indicative of poor nutritional status and/or inflammation, was significantly associated with adverse clinicopathological features, including older age, lower body mass index, deeper tumor invasion, and higher serum levels of tumor markers like CEA and CA19‐9 [55]. Most importantly, CONUT‐high patients had significantly poorer overall survival (OS) compared to CONUT‐low (≤ 3) patients with multivariate analysis (Hazard Ratio: 5.09). Time‐dependent ROC analysis further revealed that the CONUT score had a higher area under the curve (AUC) for predicting 5‐year OS than other established prognostic markers like the neutrophil‐to‐lymphocyte ratio (NLR), the modified Glasgow Prognostic Score (mGPS), or pathological stage (pStage). The CONUT score also tended to maintain its predictive accuracy for long‐term survival for an extended period post‐surgery. This highlights the CONUT score as a simple, readily available, and powerful prognostic tool in the setting of resectable GC.

### 7.2 Immune Response Markers

We has extensively investigated the programmed death‐1 (PD‐1)/programmed death‐ligand 1 (PD‐L1) pathway and its implications for cancer immunotherapy, particularly in gastrointestinal malignancies [56, 57]. Studies have assessed PD‐L1 expression and tumor‐infiltrating lymphocytes (TILs) as potential tissue biomarkers for patient selection in clinical trials targeting the PD‐1/PD‐L1 pathways in esophageal cancer [58]. We also investigated if PD‐L1 expression in biopsy samples accurately reflects its expression in the whole tumor in gastric cancer, noting that dynamic alterations by treatments might contribute to conflicting results regarding its predictive significance [59].

The prognostic impact of PD‐1 on tumor‐infiltrating lymphocytes in resected esophageal cancers has also been evaluated, including the idea of using PD‐1 expression on peripheral lymphocytes as a liquid biopsy [60]. Further research has delved into factors influencing the efficacy of PD‐1 blockade.

Fibrotic gastric cancer xenograft tumors, for instance, showed resistance to anti‐PD‐1 treatment due to increased PMN‐MDSC accumulation and decreased lymphocyte infiltration, with PDGFRα/β―blockade reversing these effects [33]. We also found that trastuzumab upregulates PD‐L1 expression through interaction with NK cells in gastric cancer [61].

Beyond PD‐1/PD‐L1, we extend to related immune pathways and factors influencing anti‐tumor responses. This includes the role of IDO1, which, similar to the PD‐1/PD‐L1 pathway, is a key player in tumor immunology and a potential therapeutic target [62]. We have also studied the prognostic and clinical impact of PD‐L2, another ligand of PD‐1, in esophageal cancers [63].

With the advent of immune checkpoint inhibitors (ICIs) in cancer treatment, new prognostic and predictive markers related to immune response are emerging. We explored the prognostic significance of immune‐related adverse events (irAEs) in patients with advanced or recurrent upper gastrointestinal cancers, including GC, who were treated with the anti‐PD‐1 antibody nivolumab [64]. Our findings were compelling: patients who developed irAEs exhibited significantly longer OS and progression‐free survival (PFS) compared to those who did not experience irAEs. This beneficial association was observed for both GC and esophageal cancer subgroups. Furthermore, the occurrence of irAEs was linked to good baseline performance status (ECOG PS) and high serum albumin levels. Multivariate analysis confirmed that the presence of an irAE was an independent favorable prognostic factor for OS. These results suggest that irAEs may reflect a robust systemic immune activation that contributes to, or is indicative of, an effective anti‐tumor immune response.

### 7.3 Hospital Volume Influencing Surgical Outcome

The standard treatment strategy for most patients with gastric cancer is surgery. Thus, it is very important to know the factors influencing surgical outcome [65]. We analyzed data from 145 523 Japanese patients between 2011 and 2015 to examine how hospital and surgeon volume affect postoperative mortality after distal gastrectomy for gastric cancer [66]. We categorized hospitals into low, medium, and high volume tiers, and surgeons into five volume groups. We found that operative mortality rates decreased significantly as both hospital and surgeon volumes increased. Specifically, operative mortality was 1.9% in low‐volume hospitals and 0.5% in high‐volume hospitals. After adjusting for patient characteristics, hospital volume remained strongly associated with better outcomes (e.g., high‐volume hospitals had an OR of 0.42). The findings highlight that hospital volume plays a crucial role in patient outcomes, suggesting that centralizing distal gastrectomy procedures could improve safety and reduce mortality. As for total gastrectomy, we categorized surgeon volume (S1‐S4) and hospital volume (H1‐H3) based on the number of TGs performed annually. We found that low‐volume surgeons and hospitals often treated older, higher‐risk patients [67]. Operative mortality decreased significantly with increased surgeon and hospital volume. Specifically, the highest volume hospitals (H3) showed an adjusted odds ratio (OR) of 0.53 for mortality compared to the lowest (H1), even after accounting for patient characteristics. This suggests that hospital volume is strongly associated with better outcomes, supporting the idea of centralizing TG procedures to improve patient safety.

### 7.4 Resistance to Chemotherapeutic Agents for Gastric and Other GI Cancers

Only a small percentage of patients with esophageal squamous cell carcinoma (ESCC) benefit from immune checkpoint inhibitors due to resistance. This highlights the need for precise predictive biomarkers for personalized immunotherapy in clinical settings.

*F. nucleatum* has been shown to contribute to chemoresistance in gastrointestinal cancers and specifically confers chemoresistance by modulating autophagy in ESCC [37]. We quantified *F. nucleatum* levels in tumor tissues from 551 ESCC patients across two cohorts [68], and found that high levels of intratumoral *F. nucleatum* were associated with significantly poorer recurrence‐free survival. Glucose transporter 1 (GLUT1) expression in pretreatment biopsy samples of esophageal cancer was associated with chemotherapy response, pathological tumor stage, and histological response grade after esophagectomy [69]. GLUT1 expression may serve as a surrogate marker for chemotherapy response. The sensitivity of gastric cancer to trastuzumab is regulated by the miR‐223/FBXW7 pathway [70]. While trastuzumab combined with chemotherapy has shown significant efficacy in HER2‐positive gastric cancer, resistance to this drug is a challenge. The efficacy of imatinib in gastrointestinal stromal tumors (GIST) is limited by resistance and toxicity, making the exploration of predictive markers for prognosis and imatinib therapeutic efficacy urgent. FBXW7 regulates imatinib sensitivity in GIST by targeting MCL1 [71]. Recent studies have elucidated critical molecular mechanisms underlying the progression and treatment resistance of GISTs [72]. The YAP/TAZ signaling pathway was shown to promote tumor growth via CCND1 activation, while its regulation through the FBXW7‐YAP axis establishes YAP as an independent prognostic marker.

Extracellular vesicles from CAFs containing Annexin A6 can induce FAK‐YAP activation by stabilizing β1 integrin, thereby enhancing drug resistance in gastric cancer. Inhibition of FAK or YAPeffectively attenuated gastric cancer drug resistance in vitro and in vivo [32]. Upregulation of ERCC1 and DPD expressions can occur after oxaliplatin‐based first‐line chemotherapy for metastatic colorectal cancer [73]. Colorectal cancer stem cells acquire chemoresistance through the upregulation of F‐Box/WD Repeat‐Containing Protein 7 (FBXW7) and the consequent degradation of c‐Myc [74].

The integration of large‐scale mult‐omics data (genomics, transcriptomics, proteomics, metabolomics, epigenomics, microbiomics) using advanced bioinformatics and artificial intelligence (AI) algorithms will be instrumental. This can lead to improved patient stratification, prediction of treatment response, identification of novel therapeutic targets, and the development of more sophisticated prognostic and predictive biomarkers.

## 8 Future Direction in Managing Gastric Cancer

Ultimately, the future of gastric cancer treatment lies in the development of highly personalized and adaptive strategies. These approaches must dynamically account for the inherent heterogeneity and evolutionary capacity of the tumor, as well as the complex and evolving interplay between cancer cells and their microenvironment. There are some research gaps to be solved in the near future, such as heterogeneity of CAFs, interaction between microbiota and immunity, and integration of multiomics data for precision oncology. The journey from understanding fundamental molecular mechanisms, as investigated by numerous researchers, to implementing effective, individualized patient care requires continued dedication to innovative research, collaborative efforts, and well‐designed clinical trials.

## Author Contributions

**Hideo Baba:** writing – original draft, writing – review and editing, project administration, supervision. **Takatsugu Ishimoto:** writing – review and editing. **Yoshifumi Baba:** writing – review and editing. **Hiromitsu Hayashi:** writing – review and editing. **Masaaki Iwatsuki:** writing – review and editing.

## Funding

The authors have nothing to report.

## Conflicts of Interest

One of the authors, Yoshifumi Baba, is an editorial member of the Ann Gastroenterol Surg. The other authors declare no conflicts of interest.

## References

[1] N. Deng , L. K. Goh , H. Wang , et al., “A Comprehensive Survey of Genomic Alterations in Gastric Cancer Reveals Systematic Patterns of Molecular Exclusivity and Co‐Occurrence Among Distinct Therapeutic Targets,” Gut 61, no. 5 (2012): 673–684. 22315472 10.1136/gutjnl-2011-301839 PMC3322587

[2] R. E. Sexton , M. N. A. Hallak , M. Diab , and A. S. Azmi , “Gastric Cancer: A Comprehensive Review of Current and Future Treatment Strategies,” Cancer Metastasis Reviews 39, no. 4 (2020): 1179–1203, 10.1007/s10555-020-09925-3. 32894370 PMC7680370

[3] Y. Ma , Z. Jiang , L. Pan , et al., “Current Development of Molecular Classifications of Gastric Cancer Based on Omics,” International Journal of Oncology 65, no. 3 (2024): 89, 10.3892/ijo.2024.5677. 39092559 PMC11302956

[4] K. Handa , R. Nakatani , Y. Hirono , T. Tanaka , H. Kunisaki , and M. Kitago , “Epigenetic Regulation in Gastric Cancer: An Update,” World Journal of Gastroenterology 26, no. 28 (2020): 4059–4072.

[5] C. Mendez‐Dorantes and K. H. Burns , “LINE‐1 Retrotransposition and Its Deregulation in Cancers: Implications for Therapeutic Opportunities,” Genes & Development 37 (2023): 948–967. 38092519 10.1101/gad.351051.123 PMC10760644

[6] X. Zhang , R. Zhang , and J. Yu , “New Understanding of the Relevant Role of LINE‐1 Retrotransposition in Human Disease and Immune Modulation,” Frontiers in Cell and Development Biology 8 (2020): 657, 10.3389/fcell.2020.00657. PMC7426637 32850797

[7] Y. Baba , N. Yasuda , M. Bundo , et al., “LINE‐1 Hypomethylation Increased Retrotransposition and Tumor‐Specific Insertion in Upper Gastrointestinal Cancer,” Cancer Science 115, no. 1 (2024): 247–256. 38013627 10.1111/cas.16007 PMC10823286

[8] Y. Baba , M. Watanabe , A. Murata , et al., “LINE‐1 Hypomethylation, DNA Copy Number Alterations, and CDK6 Amplification in Esophageal Squamous Cell Carcinoma,” Clinical Cancer Research 20, no. 5 (2014): 1114–1124. 24423610 10.1158/1078-0432.CCR-13-1645

[9] H. Shigaki , Y. Baba , M. Watanabe , et al., “LINE‐1 Hypomethylation in Noncancerous Esophageal Mucosae Is Associated With Smoking History,” Annals of Surgical Oncology 19, no. 13 (2012): 4238–4243. 22766991 10.1245/s10434-012-2488-y

[10] K. Kosumi , Y. Baba , T. Ishimoto , et al., “Relationship Between LINE‐1 Hypomethylation and *Helicobacter pylori* Infection in Gastric Mucosae,” Medical Oncology 32, no. 4 (2015): 117, 10.1007/s12032-015-0571-5. 25782871

[11] J. N. Rich , “Cancer Stem Cells: Understanding Tumor Hierarchy and Heterogeneity,” Medicine 95, no. 1 Suppl 1 (2016): S2–S7, 10.1097/MD.0000000000004764. 27611934 PMC5599210

[12] K. Eun , S. W. Ham , and H. Kim , “Cancer Stem Cell Heterogeneity: Origin and New Perspectives on CSC Targeting,” BMB Reports 50, no. 3 (2017): 117–125. 27998397 10.5483/BMBRep.2017.50.3.222 PMC5422023

[13] M. Razmi , R. Ghods , S. Vafaei , M. Sahlolbei , L. S. Zanjani , and Z. Madjd , “Clinical and Prognostic Significances of Cancer Stem Cell Markers in Gastric Cancer Patients: A Systematic Review and Meta‐Analysis,” Cancer Cell International 21, no. 1 (2021): 139, 10.1186/s12935-021-01840-z. 33639931 PMC7912890

[14] Y. Yang , W. J. Meng , and Z. Q. Wang , “The Origin of Gastric Cancer Stem Cells and Their Effects on Gastric Cancer: Novel Therapeutic Targets for Gastric Cancer,” Frontiers in Oncology 12 (2022): 960539, 10.3389/fonc.2022.960539. 36185219 PMC9520244

[15] T. Ishimoto , O. Nagano , T. Yae , et al., “CD44 Variant Regulates Redox Status in Cancer Cells by Stabilizing the xCT Subunit of System xc(−) And Thereby Promotes Tumor Growth,” Cancer Cell 19, no. 3 (2011): 387–400. 21397861 10.1016/j.ccr.2011.01.038

[16] T. Wada , T. Ishimoto , R. Seishima , et al., “Functional Role of CD44v‐xCT System in the Development of Spasmolytic Polypeptide‐Expressing Metaplasia,” Cancer Science 104, no. 10 (2013): 1323–1329. 23848514 10.1111/cas.12236 PMC7656553

[17] T. Ishimoto , H. Sugihara , M. Watanabe , et al., “Macrophage‐Derived Reactive Oxygen Species Suppress miR‐328 Targeting CD44 in Cancer Cells and Promote Redox Adaptation,” Carcinogenesis 35, no. 5 (2014): 1003–1011. 24318997 10.1093/carcin/bgt402

[18] K. Toyoshima , A. Hayashi , M. Kashiwagi , et al., “Analysis of Circulating Tumor Cells Derived From Advanced Gastric Cancer,” International Journal of Cancer 137, no. 4 (2015): 991–998. 25622566 10.1002/ijc.29455

[19] D. L. Worthley , K. D. Phillips , N. Wayte , et al., “Gastric Adenocarcinoma and Proximal Polyposis of the Stomach (GAPPS): A New Autosomal Dominant Syndrome,” Gut 61 (2012): 774–779. 21813476 10.1136/gutjnl-2011-300348

[20] J. Li , S. L. Woods , S. Healey , et al., “Point Mutations in Exon 1B of APC Reveal Gastric Adenocarcinoma and Proximal Polyposis of the Stomach as a Familial Adenomatous Polyposis Variant,” American Journal of Human Genetics 98 (2016): 830–842. 27087319 10.1016/j.ajhg.2016.03.001 PMC4863475

[21] M. Iwatsuki , C. Matsumoto , K. Mimori , and H. Baba , “The Comprehensive Review of Gastric Adenocarcinoma and Proximal Polyposis of the Stomach (GAPPS) From Diagnosis and Treatment,” Annals of Gastroenterological Surgery 7, no. 5 (2023): 725–732. 37663957 10.1002/ags3.12708 PMC10472389

[22] C. Matsumoto , K. K. Takahashi , M. Iwatsuki , et al., “Genomic and Transcriptomic Landscape of Carcinogenesis in Patients With Gastric Adenocarcinoma and Proximal Polyposis of the Stomach (GAPPS),” Proceedings of the National Academy of Sciences of the United States of America 122, no. 44 (2025): e2427133122, 10.1073/pnas.2427133122. 41171849 PMC12595452

[23] D. Hanahan and R. A. Weinberg , “Hallmarks of Cancer: The Next Generation,” Cell 144, no. 5 (2011): 646–674. 21376230 10.1016/j.cell.2011.02.013

[24] D. F. Quail and J. A. Joyce , “Microenvironmental Regulation of Tumor Progression and Metastasis,” Nature Medicine 9, no. 11 (2013): 1423–1437. 10.1038/nm.3394 PMC3954707 24202395

[25] T. Ishimoto , H. Sawayama , H. Sugihara , and H. Baba , “Interaction Between Gastric Cancer Stem Cells and the Tumor Microenvironment,” Journal of Gastroenterology 49, no. 7 (2014): 1111–1120. 24652101 10.1007/s00535-014-0952-0

[26] E. Ozmen , T. D. Demir , and G. Ozcan , “Cancer‐Associated Fibroblasts: Protagonists of the Tumor Microenvironment in Gastric Cancer,” Frontiers in Molecular Biosciences 11 (2024): 1340124, 10.3389/fmolb.2024.1340124. 38562556 PMC10982390

[27] T. Yasuda , M. Koiwa , A. Yonemura , T. Akiyama , H. Baba , and T. Ishimoto , “Protocol to Establish Cancer‐Associated Fibroblasts From Surgically Resected Tissues and Generate Senescent Fibroblasts,” STAR Protocols 2, no. 2 (2021): 100553, 10.1016/j.xpro.2021.100553. 34136831 PMC8176369

[28] F. Wei , T. Uchihara , A. Yonemura , et al., “A novel tdTomato transgenic mouse model to visualize FAP‐positive cancer‐associated fibroblasts,” FEBS Journal 290, no. 10 (2023): 2604–2615. 36565059 10.1111/febs.16712

[29] T. Ishimoto , K. Miyake , T. Nandi , et al., “Activation of Transforming Growth Factor Beta 1 Signaling in Gastric Cancer‐Associated Fibroblasts Increases Their Motility, via Expression of Rhomboid 5 Homolog 2, and Ability to Induce Invasiveness of Gastric Cancer Cells,” Gastroenterology 153, no. 1 (2017): 191–204. 28390866 10.1053/j.gastro.2017.03.046

[30] F. Kitamura , T. Semba , N. Yasuda‐Yoshihara , et al., “Cancer‐Associated Fibroblasts Reuse Cancer‐Derived Lactate to Maintain a Fibrotic and Immunosuppressive Microenvironment in Pancreatic Cancer,” JCI Insight 8, no. 20 (2023): e163022, 10.1172/jci.insight.163022. 37733442 PMC10619496

[31] M. Lou , M. Iwatsuki , X. Wu , W. Zhang , C. Matsumoto , and H. Baba , “Cancer‐Associated Fibroblast‐Derived IL‐8 Upregulates PD‐L1 Expression in Gastric Cancer Through the NF‐kappaB Pathway,” Annals of Surgical Oncology 31, no. 5 (2024): 2983–2995. 38006530 10.1245/s10434-023-14586-x

[32] T. Uchihara , K. Miyake , A. Yonemura , et al., “Extracellular Vesicles From Cancer‐Associated Fibroblasts Containing Annexin A6 Induces FAK‐YAP Activation by Stabilizing beta1 Integrin, Enhancing Drug Resistance,” Cancer Research 80, no. 16 (2020): 3222–3235. 32605995 10.1158/0008-5472.CAN-19-3803

[33] T. Akiyama , T. Yasuda , T. Uchihara , et al., “Stromal Reprogramming Through Dual PDGFRα/β Blockade Boosts the Efficacy of Anti‐PD‐1 Immunotherapy in Fibrotic Tumors,” Cancer Research 83, no. 5 (2023): 753–770. 36543251 10.1158/0008-5472.CAN-22-1890

[34] M. Amieva and R. M. Peek , “Pathobiology of *Helicobacter pylori*‐Induced Gastric Cancer,” Gastroenterology 150, no. 1 (2016): 64–78. 26385073 10.1053/j.gastro.2015.09.004 PMC4691563

[35] K. Mima , K. Kosumi , Y. Baba , T. Hamada , H. Baba , and S. Ogino , “The Microbiome, Genetics, and Gastrointestinal Neoplasms: The Evolving Field of Molecular Pathological Epidemiology to Analyze the Tumor‐Immune‐Microbiome Interaction,” Human Genetics 140, no. 5 (2021): 725–746. 33180176 10.1007/s00439-020-02235-2 PMC8052267

[36] K. Yamamura , Y. Baba , S. Nakagawa , et al., “Human Microbiome *Fusobacterium Nucleatum* in Esophageal Cancer Tissue Is Associated With Prognosis,” Clinical Cancer Research 22, no. 22 (2016): 5574–5581. 27769987 10.1158/1078-0432.CCR-16-1786

[37] Y. Liu , Y. Baba , T. Ishimoto , et al., “ *Fusobacterium nucleatum* Confers Chemoresistance by Modulating Autophagy in Oesophageal Squamous Cell Carcinoma,” British Journal of Cancer 124, no. 5 (2021): 963–974. 33299132 10.1038/s41416-020-01198-5 PMC7921654

[38] D. Nomoto , Y. Baba , Y. Liu , et al., “ *Fusobacterium nucleatum* Promotes Esophageal Squamous Cell Carcinoma Progression via the NOD1/RIPK2/NF‐kappaB Pathway,” Cancer Letters 530 (2022): 59–67. 35033591 10.1016/j.canlet.2022.01.014

[39] Y. Hara , Y. Baba , E. Oda , et al., “Presence of *Fusobacterium nucleatum* in Relation to Patient Survival and an Acidic Environment in Oesophagogastric Junction and Gastric Cancers,” British Journal of Cancer 131, no. 5 (2024): 797–807. 38992099 10.1038/s41416-024-02753-0 PMC11368944

[40] K. Mima , S. Nakagawa , H. Sawayama , et al., “The Microbiome and Hepatobiliary‐Pancreatic Cancers,” Cancer Letters 402 (2017): 9–15. 28527946 10.1016/j.canlet.2017.05.001

[41] Y. Sakamoto , K. Mima , T. Ishimoto , et al., “Relationship Between Fusobacterium Nucleatum and Antitumor Immunity in Colorectal Cancer Liver Metastasis,” Cancer Science 112, no. 11 (2021): 4470–4477. 34464993 10.1111/cas.15126 PMC8586672

[42] T. Yamane , Y. Kanamori , H. Sawayama , et al., “Iron Accelerates *Fusobacterium Nucleatum*‐Induced CCL8 Expression in Macrophages and Is Associated With Colorectal Cancer Progression,” JCI Insight 7, no. 21 (2022): e156802, 10.1172/jci.insight.156802. 36136589 PMC9675438

[43] M. Iwatsuki , K. Mimori , T. Yokobori , et al., “Epithelial–Mesenchymal Transition in Cancer Development and Its Clinical Significance,” Cancer Science 101, no. 2 (2010): 293–299. 19961486 10.1111/j.1349-7006.2009.01419.x PMC11159985

[44] M. Iwatsuki , K. Mimori , T. Fukagawa , et al., “The Clinical Significance of Vimentin‐Expressing Gastric Cancer Cells in Bone Marrow,” Annals of Surgical Oncology 17 (2010): 2526–2533. 20358301 10.1245/s10434-010-1041-0

[45] K. Mima , H. Hayashi , K. Imai , et al., “High CD44s Expression Is Associated With the EMT Expression Profile and Intrahepatic Dissemination of Hepatocellular Carcinoma After Local Ablation Therapy,” Journal of Hepato‐Biliary‐Pancreatic Sciences 20, no. 4 (2013): 429–434. 23238743 10.1007/s00534-012-0580-0

[46] N. Umezaki , S. Nakagawa , Y. Yamashita , et al., “Lysyl Oxidase Induces Epithelial‐Mesenchymal Transition and Predicts Intrahepatic Metastasis of Hepatocellular Carcinoma,” Cancer Science 110, no. 6 (2019): 2033–2043. 30919528 10.1111/cas.14010 PMC6550133

[47] Z. Liu , H. Hayashi , K. Matsumura , et al., “Hyperglycaemia Induces Metabolic Reprogramming Into a Glycolytic Phenotype and Promotes Epithelial‐Mesenchymal Transitions via YAP/TAZ‐Hedgehog Signalling Axis in Pancreatic Cancer,” British Journal of Cancer 128, no. 5 (2023): 844–856. 36536047 10.1038/s41416-022-02106-9 PMC9977781

[48] M. Kanda and Y. Kodera , “Molecular Mechanisms of Peritoneal Dissemination in Gastric Cancer,” World Journal of Gastroenterology 22, no. 30 (2016): 6829–6840. 27570420 10.3748/wjg.v22.i30.6829 PMC4974582

[49] D. Ng , D. Cyr , S. Khan , F. Dossa , C. Swallow , and K. Kazazian , “Molecular Mechanisms of Metastatic Peritoneal Dissemination in Gastric Adenocarcinoma,” Cancer Metastasis Reviews 44, no. 2 (2025): 50, 10.1007/s10555-025-10265-3. 40317360 PMC12049340

[50] Y. Kiyozumi , M. Iwatsuki , J. Kurashige , et al., “PLOD2 as a Potential Regulator of Peritoneal Dissemination in Gastric Cancer,” International Journal of Cancer 143, no. 5 (2018): 1202–1211. 29603227 10.1002/ijc.31410

[51] J. Kurashige , T. Hasegawa , A. Niida , et al., “Integrated Molecular Profiling of Human Gastric Cancer Identifies DDR2 as a Potential Regulator of Peritoneal Dissemination,” Scientific Reports 6 (2016): 22371, 10.1038/srep22371. 26934957 PMC4776110

[52] T. Yasuda , M. Koiwa , A. Yonemura , et al., “Inflammation‐Driven Senescence‐Associated Secretory Phenotype in Cancer‐Associated Fibroblastsenhances Peritoneal Dissemination,” Cell Reports 34, no. 8 (2021): 108779, 10.1016/j.celrep.2021.108779. 33626356

[53] A. Yonemura , T. Semba , J. Zhang , et al., “Mesothelial Cells With Mesenchymal Features Enhance Peritoneal Dissemination by Forming a Protumorigenic Microenvironment,” Cell Reports 43, no. 1 (2024): 113613, 10.1016/j.celrep.2023.113613. 38232734

[54] K. Takagi , P. Domagala , W. G. Polak , S. Buettner , B. P. L. Wijnhoven , and J. N. M. Ijzermans , “Prognostic Significance of the Controlling Nutritional Status (CONUT) Score in Patients Undergoing Gastrectomy for Gastric Cancer: A Systematic Review and Meta‐Analysis,” BMC Surgery 19 (2019): 129, 10.1186/s12893-019-0593-6. 31488105 PMC6729085

[55] D. Kuroda , H. Sawayama , J. Kurashige , et al., “Controlling Nutritional Status (CONUT) Score Is a Prognostic Marker for Gastric Cancer Patients After Curative Resection,” Gastric Cancer 21, no. 2 (2018): 204–212. 28656485 10.1007/s10120-017-0744-3

[56] K. Yamashita , M. Iwatsuki , J. A. Ajani , and H. Baba , “Programmed Death Ligand‐1 Expression in Gastrointestinal Cancer: Clinical Significance and Future Challenges,” Annals of Gastroenterological Surgery 4, no. 4 (2020): 369–378. 32724880 10.1002/ags3.12348 PMC7382440

[57] Y. Baba , D. Nomoto , K. Okadome , et al., “Tumor Immune Microenvironment and Immune Checkpoint Inhibitors in Esophageal Squamous Cell Carcinoma,” Cancer Science 111, no. 9 (2020): 3132–3141. 32579769 10.1111/cas.14541 PMC7469863

[58] T. Yagi , Y. Baba , T. Ishimoto , et al., “PD‐L1 Expression, Tumor‐Infiltrating Lymphocytes, and Clinical Outcome in Patients With Surgically Resected Esophageal Cancer,” Annals of Surgery 269, no. 3 (2019): 471–478. 29206673 10.1097/SLA.0000000000002616

[59] K. Yamashita , M. Iwatsuki , K. Harada , et al., “Can PD‐L1 Expression Evaluated by Biopsy Sample Accurately Reflect Its Expression in the Whole Tumour in Gastric Cancer?,” British Journal of Cancer 121, no. 3 (2019): 278–280. 31285589 10.1038/s41416-019-0515-5 PMC6738080

[60] D. Nomoto , Y. Baba , K. Okadome , et al., “Prognostic Impact of PD‐1 on Tumor‐Infiltrating Lymphocytes in 433 Resected Esophageal Cancers,” Annals of Thoracic Surgery 113, no. 1 (2022): 286–294. 33482156 10.1016/j.athoracsur.2021.01.013

[61] K. Yamashita , M. Iwatsuki , N. Yasuda‐Yoshihara , et al., “Trastuzumab Upregulates Programmed Death Ligand‐1 Expression Through Interaction With NK Cells in Gastric Cancer,” British Journal of Cancer 124, no. 3 (2021): 595–603. 33100329 10.1038/s41416-020-01138-3 PMC7851117

[62] Y. Kiyozumi , Y. Baba , K. Okadome , et al., “IDO1 Expression Is Associated With Immune Tolerance and Poor Prognosis in Patients With Surgically Resected Esophageal Cancer,” Annals of Surgery 269, no. 6 (2019): 1101–1108. 31082908 10.1097/SLA.0000000000002754

[63] K. Okadome , Y. Baba , D. Nomoto , et al., “Prognostic and Clinical Impact of PD‐L2 and PD‐L1 Expression in a Cohort of 437 Oesophageal Cancers,” British Journal of Cancer 122, no. 10 (2020): 1535–1543. 32210369 10.1038/s41416-020-0811-0 PMC7217865

[64] Y. Hara , Y. Baba , T. Toihata , et al., “Immune‐Related Adverse Events and Prognosis in Patients With Upper Gastrointestinal Cancer Treated With Nivolumab,” Journal of Gastrointestinal Oncology 13, no. 6 (2022): 2779–2788. 36636073 10.21037/jgo-22-281 PMC9830324

[65] M. Watanabe , H. Miyata , M. Gotoh , et al., “Total Gastrectomy Risk Model: Data From 20,011 Japanese Patients in a Nationwide Internet‐Based Database,” Annals of Surgery 260, no. 6 (2014): 1034–1039. 25072429 10.1097/SLA.0000000000000781

[66] M. Iwatsuki , H. Yamamoto , H. Miyata , et al., “Effect of Hospital and Surgeon Volume on Postoperative Outcomes After Distal Gastrectomy for Gastric Cancer Based on Data From 145,523 Japanese Patients Collected From a Nationwide Web‐Based Data Entry System,” Gastric Cancer 22, no. 1 (2019): 190–201. 30302654 10.1007/s10120-018-0883-1

[67] M. Iwatsuki , H. Yamamoto , H. Miyata , et al., “Association of Surgeon and Hospital Volume With Postoperative Mortality After Total Gastrectomy for Gastric Cancer: Data From 71,307 Japanese Patients Collected From a Nationwide Web‐Based Data Entry System,” Gastric Cancer 24, no. 2 (2021): 526–534. 33037492 10.1007/s10120-020-01127-8

[68] K. Yamamura , D. Izumi , R. Kandimalla , et al., “Intratumoral *Fusobacterium nucleatum* Levels Predict Therapeutic Response to Neoadjuvant Chemotherapy in Esophageal Squamous Cell Carcinoma,” Clinical Cancer Research 25, no. 20 (2019): 6170–6179. 31358543 10.1158/1078-0432.CCR-19-0318 PMC6801075

[69] H. Sawayama , Y. Ogata , T. Ishimoto , et al., “Glucose Transporter 1 Regulates the Proliferation and Cisplatin Sensitivity of Esophageal Cancer,” Cancer Science 110, no. 5 (2019): 1705–1714. 30861255 10.1111/cas.13995 PMC6500964

[70] K. Eto , M. Iwatsuki , M. Watanabe , et al., “The Sensitivity of Gastric Cancer to Trastuzumab Is Regulated by the miR‐223/FBXW7 Pathway,” International Journal of Cancer 136, no. 7 (2015): 1537–1545. 25159729 10.1002/ijc.29168

[71] Y. Koga , M. Iwatsuki , K. Yamashita , et al., “The Role of FBXW7, a Cell‐Cycle Regulator, as a Predictive Marker of Recurrence of Gastrointestinal Stromal Tumors,” Gastric Cancer 22, no. 6 (2019): 1100–1108. 30854619 10.1007/s10120-019-00950-y

[72] X. Wu , K. Yamashita , M. Lou , et al., “AT101 Suppresses Gastrointestinal Stromal Tumor Growth and Promotes Apoptosis via YAP/TAZ‐CCND1 and FBXW7‐MCL1 Axes,” Annals of Surgical Oncology 32, no. 8 (2025): 5991–6004. 40148719 10.1245/s10434-025-17247-3

[73] H. Baba , M. Watanabe , H. Okabe , et al., “Upregulation of ERCC1 and DPD Expressions After Oxaliplatin‐Based First‐Line Chemotherapy for Metastatic Colorectal Cancer,” British Journal of Cancer 107, no. 12 (2012): 1950–1955. 23169295 10.1038/bjc.2012.502 PMC3516688

[74] D. Izumi , T. Ishimoto , K. Miyake , et al., “Colorectal Cancer Stem Cells Acquire Chemoresistance Through the Upregulation of F‐Box/WD Repeat‐Containing Protein 7 and the Consequent Degradation of c‐Myc,” Stem Cells 35, no. 9 (2017): 2027–2036. 28699179 10.1002/stem.2668

